# The influence of recent social experience and physical environment on courtship and male aggression

**DOI:** 10.1186/s12862-016-0584-5

**Published:** 2016-01-21

**Authors:** Topi K. Lehtonen, P. Andreas Svensson, Bob B. M. Wong

**Affiliations:** School of Biological Sciences, Monash University, 3800 Melbourne, Victoria Australia; Department of Biology and Environmental Science, Linnaeus University, 39231 Kalmar, Sweden

**Keywords:** Aggression, Behavioural plasticity, Courtship display, Encounter rate, Environmental effect, Physiological cost, Salinity, Sexual signal, Social experience

## Abstract

**Background:**

Social and environmental factors can profoundly impact an individual’s investment of resources into different components of reproduction. Such allocation trade-offs are expected to be amplified under challenging environmental conditions. To test these predictions, we used a desert-dwelling fish, the desert goby, *Chlamydogobius eremius*, to experimentally investigate the effects of prior social experience (with either a male or a female) on male investment in courtship and aggression under physiologically benign and challenging conditions (i.e., low versus high salinity).

**Results:**

We found that males maintained a higher level of aggression towards a rival after a recent encounter with a female, compared to an encounter with a male, under low (but not high) salinity. In contrast, male investment in courtship behaviour was unaffected by either salinity or social experience.

**Conclusion:**

Together, our results suggest that male investment in aggression and courtship displays can differ in their sensitivity to environmental conditions and that not all reproductive behaviours are similarly influenced by the same environmental context.

## Background

An individual’s optimal investment in different aspects of reproduction is likely to be influenced by both social and environmental factors [1–3]. However, the way in which individuals respond to such factors is also influenced by allocation trade-offs. In particular, present investment of resources into maintenance of body condition, growth and various components of current reproduction have to be traded against each other, as well as future reproduction and survival [4]. Because environmental conditions impact the amount of available resources at any given time, such trade-offs are expected to be intensified under challenging environmental conditions [5, 6].

For reproductively active individuals, encounters with potential mates, as well as rivals, are key components of the social environment. In particular, such interactions can directly affect reproductive success by influencing both mating opportunities and the level of intrasexual competition [7]. With respect to behavioural responses, the social environment can impact both sexual displays towards members of the opposite sex and the intensity of aggression towards rivals, with these two components of reproduction often being closely linked [8]. Sexual displays and aggression often entail similar costs [9], such as loss of energy [10–14], time taken from other activities [15], and increased predation risk [16–18]. The balance between mate attraction and intrasexual aggression can also be sensitive to various aspects of the physical environment. For example, in three-spined sticklebacks (*Gasterosteus aculeatus*), the reliability of male sexual displays is compromised under eutrophied conditions, with reduced visibility from algal blooms relaxing the intensity of male-male competition that would otherwise help to maintain signal honesty [19].

Salinity can fundamentally affect a range of aquatic animals and, through its effects on species distributions, the characteristics of the ecological community as a whole [20–23]. Not only can salinity influence metabolic costs (e.g., due to challenges to osmoregulation), growth rates and egg survival [24–26] but also behavioural strategies [27, 28]. The effects of salinity on reproductive behaviours, in particular, are also highly relevant in environments where salinity levels vary temporally or spatially. For example, in the flagfish, *Jordanella floridae*, the benefits of male investment in parental care behaviour can be lower at higher salinities [29–31]. In male sand gobies, *Pomatoschistus minutus*, salinity may, in turn, affect nest-building behaviour (Lehtonen et al., in preparation), which has implications for both mate choice and male-male interactions [32, 33]. Yet, despite the apparent importance of salinity on reproductive behaviours, its direct effects on male-male interactions and sexual displays are poorly understood. Moreover, there is a lack of studies assessing the effects of the physical environment (such as salinity) and social experience on sexual behaviours contemporaneously.

The desert goby, *Chlamydogobius eremius*, is an excellent model for assessing the concomitant effects of the physical environment and social experience on investment in male-male interactions and sexual displays. The desert goby is a small (<8 cm), sexually dimorphic fish with exclusive paternal care. The species is native to both permanent (e.g., spring-fed pools) and temporary (e.g., pools fed by desert streams) bodies of water in the Lake Eyre Basin of Central Australia [34]. Water conditions in these habitats can vary extensively, especially with regard to salinity, which can range from < 5 parts per thousand (from hereon ‘ppt’) to over 100 ppt (authors’ own observations). Indeed, due to the typically high rates of evaporation and sporadic water flow/renewal in desert aquatic systems [35], desert gobies may need to be able to reproduce under a wide range of physiochemical conditions, including salinity levels. Within these environments, males must not only compete for access to a nesting resource (a crevice under rocks) and defend it aggressively against other males [36], but also attract females using elaborate courtship displays [37–39]. Both sexes are nevertheless capable of breeding repeatedly within their reproductive life cycle. We predicted that both physiological costs (e.g., increased metabolic burden from higher salinity: [40]) and recent social experience (e.g., prior exposure to a female versus male) should be important determinants of male investment in both courtship and aggression. In particular, we expected that a recent encounter with a female may decrease male investment in costly courtship due to an increase in the rate of perceived mating opportunities as compared to a rival encounter. In this respect, desert gobies have earlier been found to strategically adjust their courtship effort in relation to female phenotype [39], especially in terms of body size [37], with female encounter rate being important in mediating male behaviour [38]. Here, we were interested in testing how male investment in courtship is affected by the sex of the recently encountered conspecific. We expected that, under challenging conditions, males are able to make a lower resource allocation to behavioural plasticity and, therefore, strategic adjustment of courtship effort (if any) should be less pronounced under the physiological cost of a higher salinity level [40].

We also expected males to be strategic with regard to their investment in male-male aggression. In this regard, we were interested in determining whether investment in aggression and sexual displays follow a similar pattern. In particular, previous studies have found contrasting evidence in terms of whether or not a recent female encounter increases the investment in male-male interactions [36, 41–43]. Hence, we set out to clarify the issue by assessing the effect of the type of social encounter (male vs. female) on male investment in both courtship and aggression. Because both social and salinity conditions vary within and among desert goby populations in the wild, we were also interested in any interaction effects between recent social experience and salinity. For example, harsher environments may reduce sensitivity to the social environment if plastic responses to the latter are costly.

## Methods

### Fish collection and housing

The laboratory-based experimental trials of this study took place from October 2009 to April 2010. Desert gobies used in the experiment were first generation laboratory-born individuals, whose parents were collected as juveniles from waterholes and springs located within the northern genetic grouping of *C. eremius* [35] west of Lake Eyre in South Australia. The individuals were from several different families and had been housed in 80–250 litre aquaria, separated by sex after maturation. These holding aquaria had a fine gravel substrate with halved flowerpots and plastic plants for cover. The tanks were maintained at a temperature of 23–26 °C, a salinity of approx. 6 ppt and on a 12:12 h light:dark cycle. During this time, fish were fed 1–2 times a day on a diet of commercial fish food pellets and frozen brine shrimp (*Artemia*).

### Measurement of fish weight

Immediately before being used in the experiment, fish were individually photographed by placing them into a shallow container of water (3 cm depth) with 2 mm grid lines on the bottom for scale. Fish were then weighed to the nearest 0.01 g in a container of water on an electronic balance.

### Acclimatisation of focal males to different salinities

**Fig. 1:**
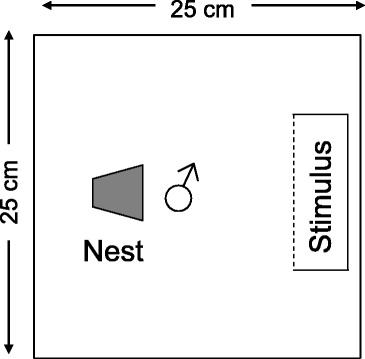
Top-view of the experimental set-up

At the start of each replicate, a male desert goby was introduced into an experimental arena with a 3 cm layer of sand on the bottom and a halved clay flowerpot (diameter: 6.5 cm; length: 6.5 cm) as a nesting resource. The experimental arena measured 25 cm × 25 cm × 20 cm (length in the direction of nest entrance × width × water depth) with the nest entrance facing the stimulus compartment (Fig. 1, see below). For logistic reasons (tank availability), we had to run our replicates in several batches over the course of the study. In most batches, we ran 1 replicate of each treatment (range: 0–2), with each concomitant replicate being subjected to the same acclimatisation schedule. In particular, we gradually (over ca. 24 h) increased salinity in each tank from their initial salinity of 6 ppt to approx. 18–20 ppt (19.0 ± 0.1, *n* = 78 salinity measurements). We then gave the males several days (4.8 ± 0.2, *n* = 90) to acclimate to these conditions. Salinity was then either (i) gradually reduced to approx. 5 ppt (5.2 ± 0.04, *n* = 44 salinity measurements) in the low salinity treatment (see below) or (ii) further increased to approx. 35 ppt (35.7 ± 0.1, *n* = 46) in the high salinity treatment. These changes in salinity levels were made so that all fish, irrespective of treatment, were subjected to a substantial change in salinity. Finally, the focal males were then allowed acclimate to the target salinity for ~ 1 week (6.7 ± 0.3 days, *n* = 90) before the onset of the experimental trials. Each focal male was only used once and therefore experienced only one salinity treatment.

We only used sexually mature females, as determined by their distended bellies, and reproductively active males, as determined by their bright adult coloration. Individuals were randomly distributed among the different treatments.

### Experiment 1: courtship

The aim of experiment 1 was to investigate the effects of salinity and social experience on male investment in courtship behaviour. For this purpose, we employed a factorial design with two factors: salinity (low and high) and recent social experience (female or male encounter), resulting in four treatment combinations. Each focal male was exposed to only one treatment. The sample sizes and body mass of focal males used in each treatment are given in Table 1.

**Table 1:** The sample size and average body mass of the focal male in each experiment

	Experiment 1-courtship				Experiment 2-aggression			
Social experience	Male		Female		Male		Female	
Salinity	Low	High	Low	High	Low	High	Low	High
Sample size	11	12	11	12	11	11	11	11
Weight (g) ± SE	3.3 ± 0.4	2.5 ± 0.2	2.7 ± 0.3	2.7 ± 0.2	2.8 ± 0.3	2.8 ± 0.3	2.8 ± 0.4	3.0 ± 0.3

In the first phase of the experiment (‘prior experience’), we manipulated the recent social experience of the focal males. For this purpose, we placed a clear container (5 cm × 15 cm; water depth: 22 cm) inside the front part of the experimental arena (Fig. 1). Ten minutes later, we added the first stimulus individual, either a female or male into the container, depending on treatment. Salinity in the stimulus container was 6 ppt, corresponding to the salinity in which the stimulus individuals had been maintained in the laboratory. One hundred minutes later, the social experience phase was ended by removing the container with the stimulus fish.

Approximately thirty minutes after completion of the first phase, the second phase (i.e., courtship measurement phase) was initiated by adding the stimulus female in the container. The procedures for presenting stimulus individuals and collecting data on focal fish behaviour were slightly modified from previously published methods [37–39]. In particular, three and a half minutes after addition of the stimulus female, and thereafter every 4 min, the behaviour of the focal nest-holder was observed for 25 s on 24 different occasions. Hence, the duration of the observation period was 24 × 4 min = 96 min. The tanks were brightly lit from above, with the observer seated in the dark away from the tank to prevent disturbance to the fish. The focal male was recorded as courting the stimulus female if he was within 5 cm of her compartment, with his body oriented towards her whilst engaged in courtship behaviour (i.e., fin displays, ‘hopping’ displays, or ‘leads towards the nest’; *sensu* [38, 39]). Both ‘the total time used for a behaviour’ and the number of ‘bouts of a behaviour’ are relevant and widely used measures of sexual behaviour [44]. Accordingly, in the analyses we quantified courtship behaviours in two different ways: (1) the proportion of the observed time that the focal male performed the behaviour, and (2) the number of distinct bouts of courtship during the observed time.

### Experiment 2: aggression

The aim of this experiment was to investigate how salinity and social experience influence the investment in aggression directed towards rival males. In particular, the experimental procedures were otherwise identical to those of experiment 1, except that the stimulus individual in the data collection phase was a male instead of a female. We quantified the intensity of aggression the focal male directed towards the stimulus male in two different ways: (1) the time spent in aggressive behaviours (fin displays and attacks), and (2) the number of aggression bouts (bursts of fin displays and number of attacks).

Fish were never used more than once as a stimulus or focal individual. However, in total 20 males (randomly distributed among the treatments) were used once in both roles, in order to decrease the number of individuals needed for the experiment.

### Statistical analyses

All statistical analyses were conducted using the software R 3.0.2 (R-Development-Core-Team 2014). We fitted models with our two treatments, *prior experience* (male/female) and *salinity* (low/high), and the interaction of the two, as main effects, and focal male size and stimulus fish size (and their interaction) as covariates. A minimal adequate model was achieved by stepwise model selection (α = 0.05; [45]). One challenge with this method is how to treat near-significant interactions. Accordingly, if the interaction between the two treatments approached significance (*p* < 0.15), we analyzed, as a conservative approach, the two treatments as a single, four-level factor, followed by Tukey’s post hoc test. Due to the potential limitations of traditional stepwise model selection, we also performed Bayesian model averaging to confirm that we had correctly identified important factors. The Bayesian analysis was carried out with the BMA 3.13 R package [46], and allowed us to analyze fully factorial models (i.e., with both treatments, focal and stimulus individual sizes, and all possible interactions). This method calculates a posterior probability of inclusion (Pr_inc_) for each main effect and interaction. Pr_inc_ is the probability that the predictor has a non-zero coefficient in the model, and is used as a measure of the influence of that predictor on the response variable. Although thresholds are not used in Bayesian statistics, values of Pr_inc_ below 0.5 can be considered as “no evidence”, and between 0.5 and 0.75 as “weak evidence” [47]. To improve residual normality and homoscedasticity in models analyzing proportions of time, we used arcsine square-root transformation [45]. Bout number was square-root transformed when this was needed to address the parametric assumptions of the model.

## Results

### Experiment 1: courtship

**Fig. 2:**
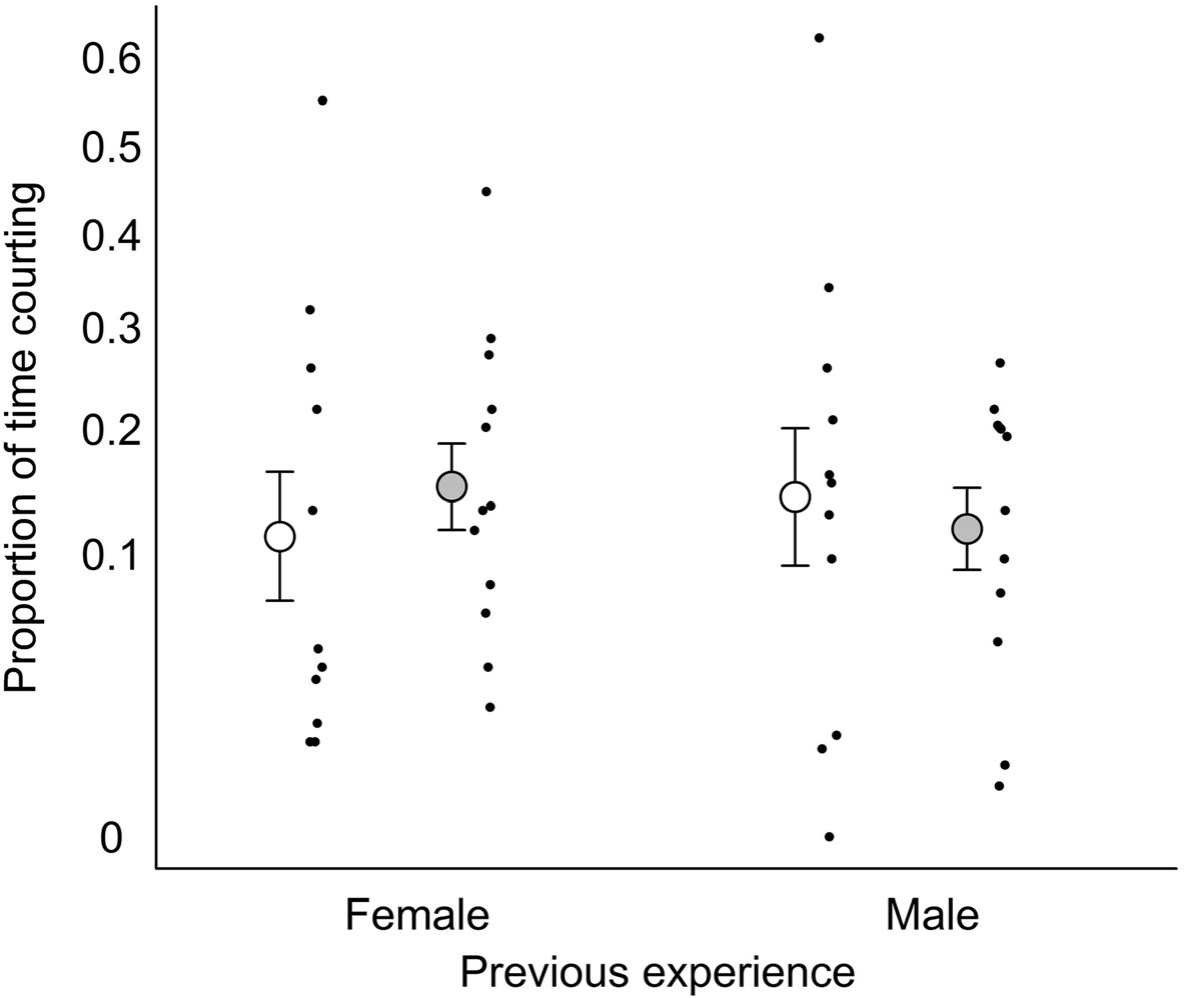
The proportion of time focal males spent courting a stimulus female after recent social experience with either a female or a male. White and grey circles with error bars indicate low and high salinity, respectively (mean ± SE). Black dots show individual focal males. Y-axis has arcsine square root scale

We investigated the proportion of time spent courting by fitting a factorial model with social experience, salinity and their interaction as main effects, and female and male mass as covariates. There was a non-significant trend for males to bias their courtship towards large females (General linear model: F_1,41_ = 2.18, *p* = 0.10), but no other main effects or interaction were significant (General linear model: *p* > 0.78: Fig. 2). Bayesian model averaging of a full factorial model confirmed these results: female size had a low probability of inclusion (P_inc_ = 25 %) and all other factors had even lower probabilities (P_inc_ < 8 %).

**Fig. 3:**
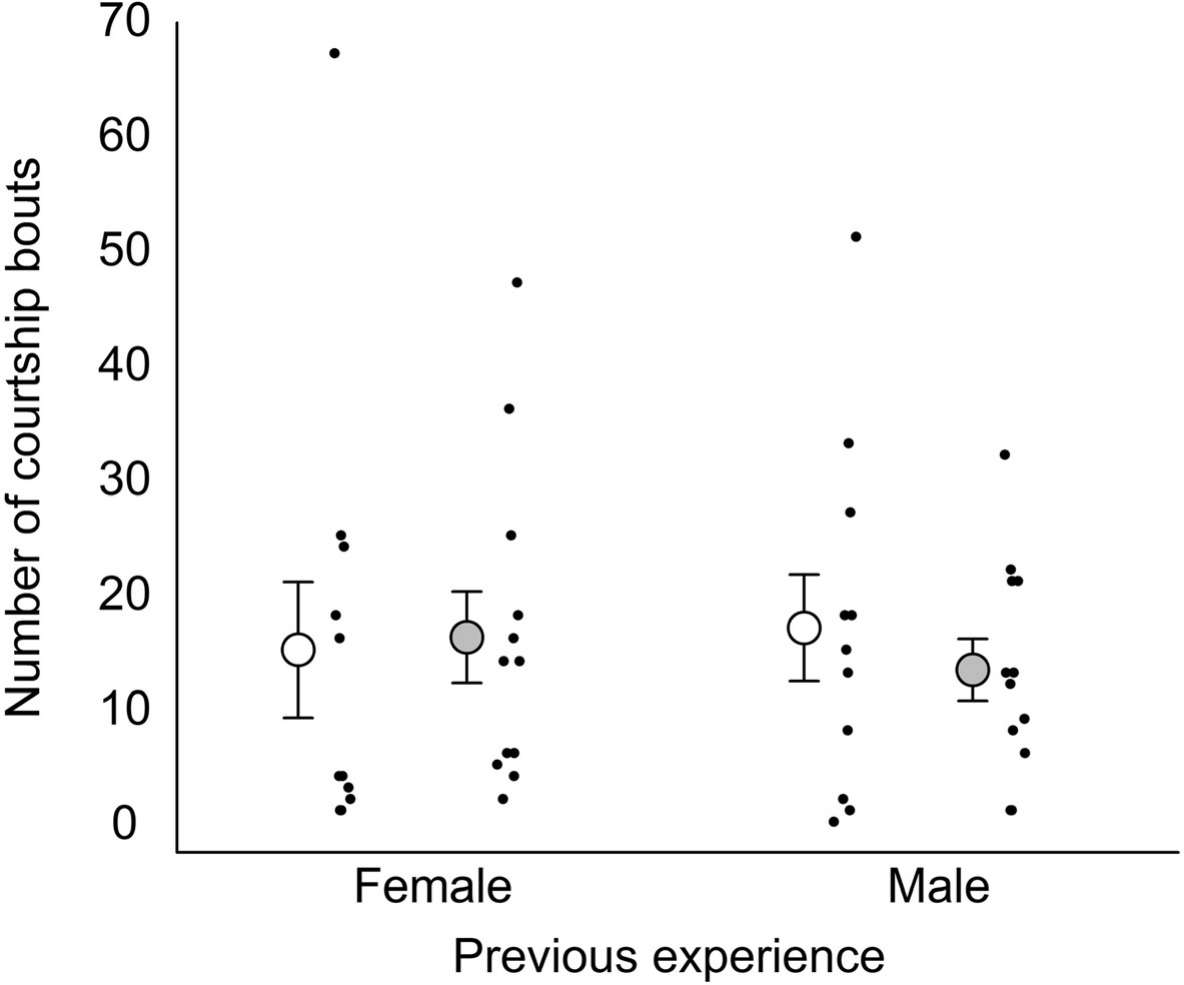
Number of courtship bouts after recent social experience with either a female or a male. White and grey circles with error bars indicate low and high salinity, respectively (mean ± SE). Black dots show individual focal males

In terms of the number of courtship bouts, stepwise selection of models with fish sizes as covariates did not identify any main effects or interactions as significant (General linear model: *p* > 0.16; Fig. 3). Similarly, Bayesian model averaging of a full model did not identify any important factors (all P_inc_ < 6.9 %).

### Experiment 2: aggression

**Fig. 4:**
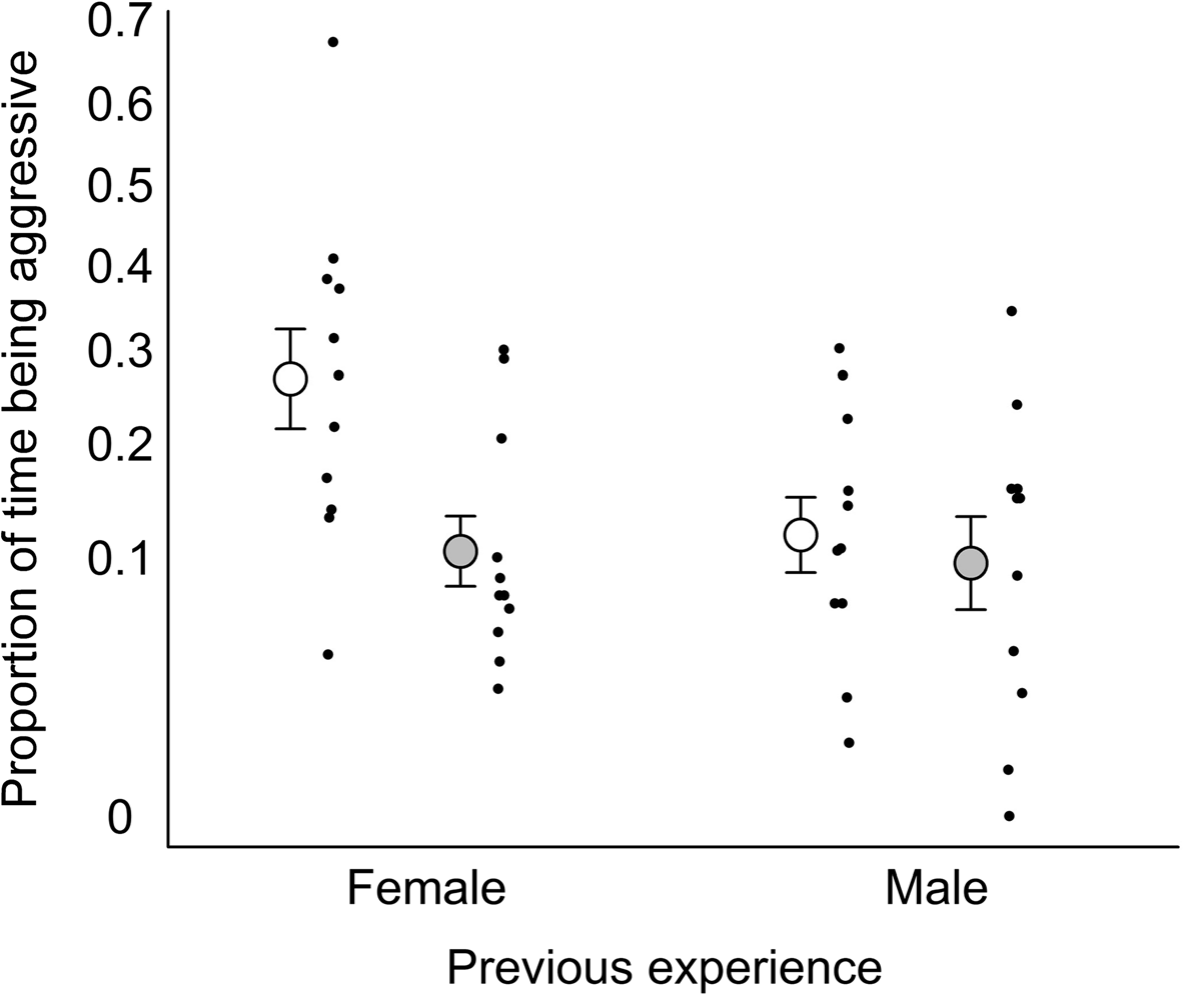
The proportion of time focal males invested in aggression after recent social experience with either a female or a male. White and grey circles with error bars indicate low and high salinity, respectively (mean ± SE). Black dots show individual focal males. Y-axis has arcsine square root scale

Stepwise model selection revealed that none of the covariates or interactions had significant effects on the proportion of time males were aggressive (General linear model: *p* > 0.13). However, the time being aggressive was significantly lower in high salinity (General linear model: F_1,41_ = 5.3, *p* = 0.027; Fig. 4). Prior social experience also had a near significant effect, with a trend that males who had experienced a female in phase 1 being more aggressive to the intruder male compared to males who had experienced a male (General linear model: F_1,41_ = 3.7, *p* = 0.061).

Because the interaction between salinity and social experience had a near significant effect on the proportion of time being aggressive (General linear model: F_1,40_ = 2.8, *p* = 0.099; Fig. 4), the data were further analysed with the two treatments combined to a four level factor, with the male body sizes as covariates. In this analysis, male sizes and all interactions were also non-significant (General linear model: *p* > 0.15). However, there was an effect of the four-level factor ‘salinity-social experience’ on aggression (General linear model: F_1,40_ = 4.5, p = 0.008). Post-hoc testing with Tukey style contrasts revealed that males in low salinity that had previously seen a female, spent more time being aggressive than such males in high salinity (Tukey’s test: *p* > 0.033; Fig. 4). In addition, within the low salinity treatment, males previously exposed to a female had a non-significant trend to be more aggressive than males exposed to another male (*p* > 0.063). No other contrasts were significant (Tukey’s test: *p* > 0.96). Bayesian model averaging of a full factorial model corroborated these results by identifying salinity as the most important factor for the proportion of time males were aggressive (P_inc_ = 64 %; all other factors P_inc_ < 39 %).

**Fig. 5:**
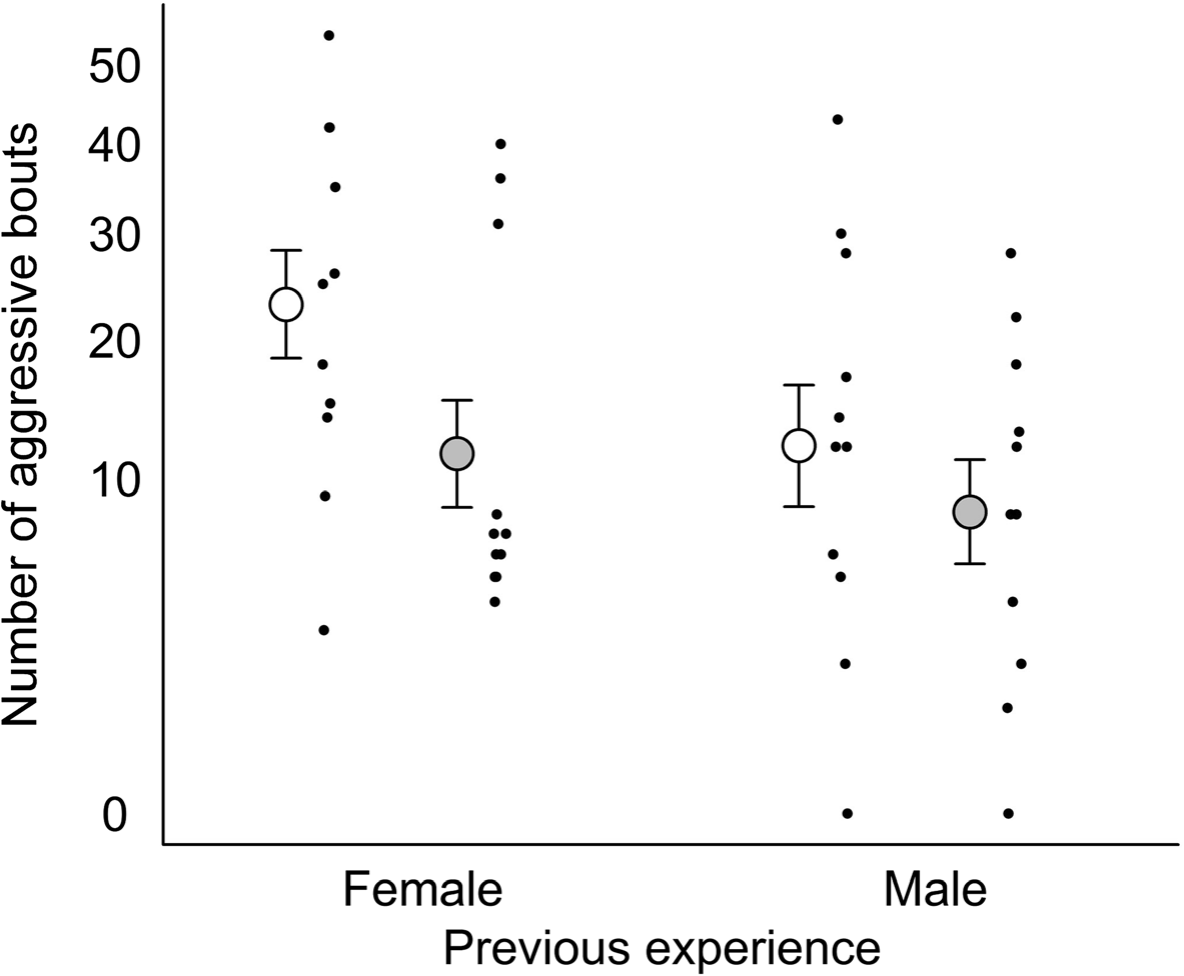
Number of aggression bouts after recent social experience with either a female or a male. White and grey circles with error bars indicate low and high salinity, respectively (mean ± SE). Black dots show individual focal males. Y-axis has square root scale

Regarding the number of bouts of aggression, stepwise selection revealed that the covariates and interactions were non-significant (*p* > 0.10). There was a trend for a higher number of aggressive bouts in high salinity (General linear model: F_1,41_ = 3.8, *p* = 0.056; Fig. 5). There was also a trend that males who had experienced a female in phase 1 performed more aggressive bouts compared to males who had experienced a male (General linear model; F_1,41_ = 3.3, *p* = 0.076). Bayesian model averaging of a fully factorial model identified neither treatment as important (P_inc_ < 39 %). Thus, the number of aggressive bouts showed similar but weaker patterns compared to the proportion of time (above).

## Discussion

We assessed how both male courtship displays and male-male aggression are affected by salinity (a key environmental factor for many aquatic species) and recent social encounter with either a rival or potential mate. We found some support for our prediction that environmental and social factors influenced sexual behaviour in desert gobies. Notably, male exhibited aggression at a lower level in a high than low salinity, and this overall difference was mostly driven by a lower level of aggression after having encountered a female (potential mate) under high salinity conditions (Fig. 4). In other words, the recent experience of encountering a female (relative to encountering a male) increased aggression towards a rival under low (but not high) salinity. In contrast to aggression, however, the investment in courtship behaviour was unaffected by either salinity or social experience.

We predicted that male desert gobies would adjust their courtship to their social and physical environment. Why, then, was this not the case? Regarding the social environment, we were interested in testing how courtship investment is affected by the sex of a recently encountered individual, rather than comparing different levels of overall social exposure *per se*. Because of this design, however, it remains possible that prior exposure to a male and female had a very similar effect, resulting in no significant difference between the two treatments. In particular, a perceived presence of a rival male has been found to result in a decreased investment in courtship is some other fish species [48–50], whereas a higher perceived rate of female encounters in desert gobies resulted in a lower male courtship investment (at least towards females of a lower quality; *sensu* [38]). In other words, both social exposure treatment levels may have resulted in a lower level of courtship compared to no recent social experience at all.

We also found no effect of a potentially more demanding physiological environment (high salinity level) on male courtship. Specifically, despite the known metabolic costs of higher salinity levels on desert gobies [40], males performed courtship behaviours unaffected by salinity. We offer the following, mutually non-exclusive hypotheses for this result. First, males may be well adapted to perform normal courtship behaviours under a wide range of different salinities. However, performing a similar level of courtship irrespective of salinity may also have involved a higher current investment under the high salinity treatment − potentially at the expense of future condition. Manipulations of body condition could offer a way to address this possibility in the future. In the current study, however, all males were well fed and in a good condition. It therefore remains possible that the lack of a significant courtship adjustment to the environmental cues were a suboptimal response under those conditions (*sensu* ‘evolutionary trap’ [51, 52]). Another way of addressing this possibility in the future would be to assess courtship of desert gobies exposed to salinities that are too high for successful reproduction (e.g., due to challenges to sperm or egg performance). In the current study, we deliberately exposed males to significant salinity changes a week prior to the assessments in both salinity treatments, with ca. 35 ppt being the highest salinity level experienced by the males. This design did not, however, assess whether males experiencing longer term, stable differences in salinity conditions would eventually have started to exhibit significant differences in their courtship investment. Finally, it remains possible that salinity (or prior social experience) does have effects on courtship but that these were too slight to be detected with the sample sizes used in this study.

In contrast to courtship, and in support of our prediction, the rate of aggression towards a stimulus rival was reduced in high salinity. Why were the findings, in this respect, different for courtship and aggression? One possibility is that the level of aggression could be more easily adjusted compared to sexual signalling. For example, aggression has been found to be highly flexible in relation to previously gathered information [53–56]. The level of aggression can also be particularly sensitive to costs, with aggressive behaviour commonly resulting in a significant loss of energy [10–13, 41] and a risk of injury, or even death [11, 57–59]. Besides simple energy depletion, aggression and fighting may also result in other negative metabolic consequences, such as elevated oxygen consumption [41] and the accumulation of lactate and other metabolic products [10]. Therefore, compared to courtship, it is possible that aggressive displays are more costly to perform, especially in demanding environmental conditions, with high salinity environments having been shown to represent significant physiological costs to many species [24], including the desert goby [40]. Such costs, in turn, are important in the context of investment in contests e.g., in the house cricket, *Acheta domesticus* [41] and cichlid fish *Tilapia zillii* [11]. Finally, it remains possible that the males perceived the experimental setup as an environment with abundant nesting opportunities (due to no competition at the time of claiming a nesting resource), but scarce in female encounters, which could then have resulted in the males enduring the costs of the high salinity environment when courting females, but not when being aggressive towards males.

Intriguingly, the level of aggression was higher after a previous encounter with a female (than after encountering a male) in low, but not high, salinity (Fig. 4). The higher level of aggression in the low salinity is in accordance with earlier studies in other taxa, such as insects and spiders, in which encounters with, or presence of, females have been found to increase male fighting effort [41–43, 58]. We note that in an earlier study on desert gobies, we did not find a previous female encounter to affect male investment in aggression when compared with no social encounter [36]. Apart from differences in experimental set-up between the studies (e.g., in terms of female encounter rate, types of social experience, and replicate duration), the type or size of the nesting resource used in each study could also be relevant for flexibility of aggression, if it affects the nesting resource’s value, as perceived by the nest-holding male.

Earlier studies suggest that body size is often relevant both in the context of courtship displays and male aggression. For example in three-spine sticklebacks, *Gasterosteus aculeatus*, an increased size difference between interacting individuals resulted in increased levels of aggression and decreased rates of courtship displays [60]. In addition, although larger males are often more dominant in competitive interactions in many species, sometimes small individuals may compensate by initiating aggression more quickly [61, 62], as seen also in desert gobies [36]. In this respect, body size may also impact the endurance of males in courtship or contests [63]. Accordingly, we included body sizes of both the focal male and stimulus individual as covariates in our analyses. The results indicate that males had a non-significant tendency to invest more in courting large, presumably more fecund, females. This result is in line with earlier findings showing that desert goby males strategically adjust courtship towards larger females [37, 39], but only when females are encountered simultaneously or in quick succession, i.e., the perceived female encounter rate is high [38].

## Conclusion

To conclude, we found that a potentially harsher environment can have different effects on sexual and aggressive displays. In particular, although neither salinity nor previous sexual experience affected the investment in courtship, desert goby males used less time for aggressive interactions with a rival under high salinity conditions. In addition, under low salinity, social experience was important, with the level of aggression being higher after encountering a female. Hence, the results suggest that it is important to consider social experience as a factor impacting aggressive behaviour (see also e.g., [64]). Moreover, our findings indicate that the investment in aggression could be more sensitive to environmental conditions than investment in sexual displays. In this respect, the result that courtship was unaffected by environmental factors indicated that desert gobies─which are often exposed to variable environmental conditions in the wild─have maintained their eagerness to perform a normal rate of sexual displays under a range of salinity levels. The lower investment in aggression under the higher salinity level, in turn, may either be an adaptive plastic response, especially if rivals are also more likely to be sluggish in such an environment, or it may represent a failure to react appropriately to a threat posed by another male, which could potentially result in negative fitness consequences. Future studies are needed to disentangle between these intriguing alternatives.

## Ethics

This study complies with all the relevant Federal and State laws of Australia, and was conducted under ethics permit (no. BSCI/2007/12) from the Biological Sciences Animal Ethics Committee of Monash University.

### Availability of supporting data

Our data-set has been uploaded to Dryad: http://dx.doi.org/10.5061/dryad.m34dv.
